# The Prognosis Prediction Model for Endometrial Cancer Based on DNA Methylation Signature

**DOI:** 10.1002/cnr2.70218

**Published:** 2025-06-17

**Authors:** Ran Ran, Ming Wang, Jinwei Miao

**Affiliations:** ^1^ Department of Obstetrics Beijing Youan Hospital, Capital Medical University Beijing China; ^2^ Department of Gynecologic Oncology Beijing Obstetrics and Gynecology Hospital, Capital Medical University. Beijing Maternal and Child Health Care Hospital Beijing China

**Keywords:** DNA methylation, endometrial cancer, prognosis, targeted treatment

## Abstract

**Background:**

DNA methylation alteration is a common event during the carcinogenesis and progression of endometrial cancer (EC). Our study aimed to investigate the value of DNA methylation‐related genes in predicting the prognosis and immunotherapy response for EC patients.

**Methods:**

The clinical information and the expression of DNA methylation‐related genes of 544 endometrial cancers were obtained from the Cancer Genome Atlas (TCGA) database. The univariate Cox regression analysis and the LASSO regression analysis were subsequently used to identify prognosis‐related methylation regulators and construct a risk model. Gene functional enrichment analysis, immune infiltration analysis, drug sensitivity analysis, and molecular feature analysis were performed in different subgroups.

**Results:**

25 methylation‐Related gene signatures were found in EC patients and are correlated to tumor differentiation and tumor metastasis. By LASSO‐Cox regression analyses, a recurrence prediction model and a prognostic‐related model were constructed based on methylation‐related genes in the TCGA training cohort. The Area Under the Curve (AUC) values of the recurrence prediction model were 0.671, 0.708, and 0.689 for 1‐, 3‐, and 5‐year time points, respectively, while those of the prognostic model were 0.731, 0.717, and 0.725. The relationship of risk score (RS) with ER/PR‐related genes, immune checkpoint expressions, and IC50s of paclitaxel, cisplatin, tamoxifen, and cetuximab was investigated. The results showed That patients in the low‐risk group are more effective in cetuximab and immune checkpoint blockade (ICB) treatment.

**Conclusions:**

The model based on the methylation‐related genes showed promising outcomes in predicting the recurrence and treatment response of EC. The patients with high‐risk scores showed a poorer prognosis and may benefit more from the treatment of cetuximab or immune checkpoint inhibitors.

**Abbreviations**
AUC: Area Under the Curve
CDF: cumulative distribution function
CN‐H: copy number high
CN‐L: copy number low
DCA: Decision Curve Analysis
EC: endometrial cancer
EH: endometrial hyperplasia
GDSC: Genomics of Drug Sensitivity in Cancer
ICB: immune checkpoint blockade
MSI‐H: microsatellite instability‐high
PFS: progression‐free survival
PPI: The protein–protein interaction
ROC: receiver operating characteristic
RS: risk score
SD: standard deviation
TCGA: The Cancer Genome Atlas
TIICs: tumor‐infiltrating immune cells
TME: tumor microenvironment

## 1 Introduction

Endometrial cancer (EC) is the most common gynecologic malignancy, accounting for a significant proportion of cancer‐related deaths among women worldwide, with an estimated 76 000 deaths annually [1, 2]. The early diagnosis of EC at the early stage and finding new biomarkers for the management of EC is the key to reducing the advanced, metastatic, or recurrent disease [3, 4].

Recent advancements in molecular profiling of endometrial cancer progression and treatment response offer new avenues for its early diagnosis, including TCGA molecular classification, L1CAM, and CTNNB1 mutation [3, 4, 5, 6, 7]. Endometrial cancer was classified into 4 subtypes based on the mutation burden and copy number alteration, which include microsatellite instability‐high(MSI‐H), copy number high(CN‐H), ultramutation, and copy number low(CN‐L) [3, 4]. Patients with different subtypes showed distinct prognoses and sensitivities to chemotherapy and immune checkpoint inhibitors, which are widely used in the clinical management of endometrial cancer [3, 4]. However, these classifications exhibit limitations in clinical utility. For instance, the majority of EC cases fall into the CN‐L subtype, which lacks distinct molecular drivers, leading to ambiguous prognostic guidance [6].

Additionally, biomarkers like L1CAM, though associated with recurrence, demonstrate variable sensitivity and fail to predict responses to emerging therapies such as immune checkpoint inhibitors (ICIs) [5].

DNA methylation, a key epigenetic regulator, has emerged as a promising avenue for refining cancer prognostication. Hypermethylation of tumor suppressor genes and hypomethylation of oncogenes drive carcinogenesis across malignancies, including EC [7, 8, 9]. In CN‐L EC, aberrant methylation patterns may dominate tumor progression, yet their prognostic and therapeutic implications remain underexplored [10, 11, 12, 13]. Existing studies on methylation in EC are fragmented, focusing on isolated genes (e.g., RASSF1, CDH13) without integrating multi‐gene signatures into predictive models [13, 14]. Moreover, the interplay between methylation‐driven pathways and tumor microenvironment (TME) remodeling—critical for immunotherapy response—has not been systematically evaluated.

This study aims to develop a prognostic model for EC based on previously reported altered DNA methylation genes of EC, which not only predicts recurrence but also identifies potential therapeutic targets, thereby addressing a critical gap in current clinical practice (Table S1).

## 2 Materials and Methods

### 2.1 Datasets and Methylation‐Related Genes

We conducted a systematic literature review to identify studies evaluating DNA methylation changes as biomarkers for the early diagnosis of EC. We searched PubMed, Embase, and the Cochrane Library for all relevant English‐language articles published before August 23, 2023. The search utilized the keywords “Endometrial Neoplasms” and “DNA Methylation.” Our review identified 25 individual genes as potential biomarkers, including CDH13, HOXA9, ADCYAP1, ASCL2, HAND2, BHLHE22, GTF2A1, CELF4, HS3ST2, HSPA2, CADM1, CDO1, GALR1, HAAO, HTR1B, MAGI2, MAL, MME, NPY, POU4F3, PCDHGB7, RASSF1, TBX5, ZNF662, and ZNF454 [14].

### 2.2 The Protein–Protein Interaction (PPI) Analysis in EC

PPI network corresponding to the previously mentioned differentially expressed genes (DEGs) was developed using the Search Tool for the Retrieval of Interacting Genes (STRING, available at https://string‐db.org/) [15].

### 2.3 Non‐Negative Matrix Factorization (NMF) Clustering Algorithm

The RNA‐sequencing expression (level 3) profiles and corresponding clinical information for EC were downloaded from the TCGA dataset(https://portal.gdc.com)which include 177 normal tissues and 544 tumor tissues. Consistency analysis by using the ConsensusClusterPlus R package (v1.54.0) indicates that the maximum number of clusters is 6, and 80% of the total sample is drawn 100 times, clusterAlg = “hc”, innerLinkage = ‘ward.D2’. Use the R software package pheatmap (v1.0.12) for clustering heatmaps. The gene expression heatmap retains genes with SD > 0.1. If the number of input genes is more than 1000, it will extract the top 25% of genes after sorting the SD.

The “proportion of ambiguous clustering” (PAC) measure quantifies the middle segment. It is defined as the fraction of sample pairs with consensus indices falling in the interval (u1, u2) ∈ [0, 1], where u1 is a value close to 0 and u2 is a value close to 1 (for instance u1 = 0.1 and u2 = 0.9). A low value of PAC indicates a flat middle segment, and a low rate of discordant assignments across permuted clustering runs. We can infer the optimal number of clusters by the K value having the lowest PAC.

The expression levels of 25 genes were compared across normal and tumor tissues, patients with different differentiation stages (G1, G2, G3), early differentiation stages (IA, IB, IC) and patients between stage IA1 and IIIC.

### 2.4 Prognostic Analysis and Construction of a Nomogram

Cox regression analyses (univariate and multivariate) were performed using R's survival package to explore the links between prognosis, risk scores, and clinical factors like age and grade. Based on the prognostic signature of methylation‐related genes and various clinical parameters, a nomogram was developed to predict the overall survival and progression‐free survival probability of patients with EC. The predictive accuracy of the nomogram was evaluated by juxtaposing the estimated survival probabilities with the ROC curves. Calibration curves for 1‐, 3‐, and 5‐year survival intervals were constructed utilizing the “rms” package in R.

### 2.5 Relationship Between Methylated Genes and Different Drug Treatment

A correlation map of various genes, including estrogen and progesterone receptors, was created with the R package ggstatsplot. Furthermore, a multi‐gene correlation heatmap was generated utilizing the pheatmap package in R. Spearman's correlation analysis was conducted to elucidate the relationships among quantitative variables that deviate from a normal distribution. Statistical significance was established for *p* values less than 0.05 (*p < 0.05).

Hormone therapy and chemotherapy response for each sample was predicted using the Genomics of Drug Sensitivity in Cancer (GDSC) database, which is the largest public pharmacogenomics resource. The prediction process was conducted using the R package “pRRophetic.” The IC50 of the samples was estimated using ridge regression with default settings. The ComBat method adjusted for batch effects, considering tissue types. Duplicate gene expressions were averaged.

### 2.6 Analysis of Tumor Immune Cells and Immune Checkpoint

The TIMER, MCPOUNTER, and CIBERSORT algorithms were employed to evaluate the distributions of tumor‐infiltrating immune cells (TIICs) about variations in the risk score, respectively. The expression of immune checkpoint‐related genes ITPRIPL1, SIGLEC15, TIGIT, CD274, HAVCR2, PDCD1, CTLA4, LAG3, and PDCD1LG2 was analyzed to predict the response to immune checkpoint blockade (ICB) therapy using the TIDE algorithm.

### 2.7 Statistical Analysis

R software (version 4.3.2) was utilized for the statistical analyses. The correlation analysis was performed using the Pearson method via the “corrplot” R package. The difference between the two groups was compared by the Mann–Whitney U test. The “survival” R package was used to perform univariate and multivariate Cox hazard regression analyses. The Kaplan–Meier curve was employed to compare the survival difference by the log‐rank test. A nomogram predicting EC patient survival was created and validated with K‐fold cross‐validation. The calibration curve was utilized to illustrate the predictive efficacy of the nomogram. *p* < 0.05 was considered statistically significant.

## 3 Results

### 3.1 Defining the Expression of Methylation‐Related Gene in EC

**FIGURE 1:**
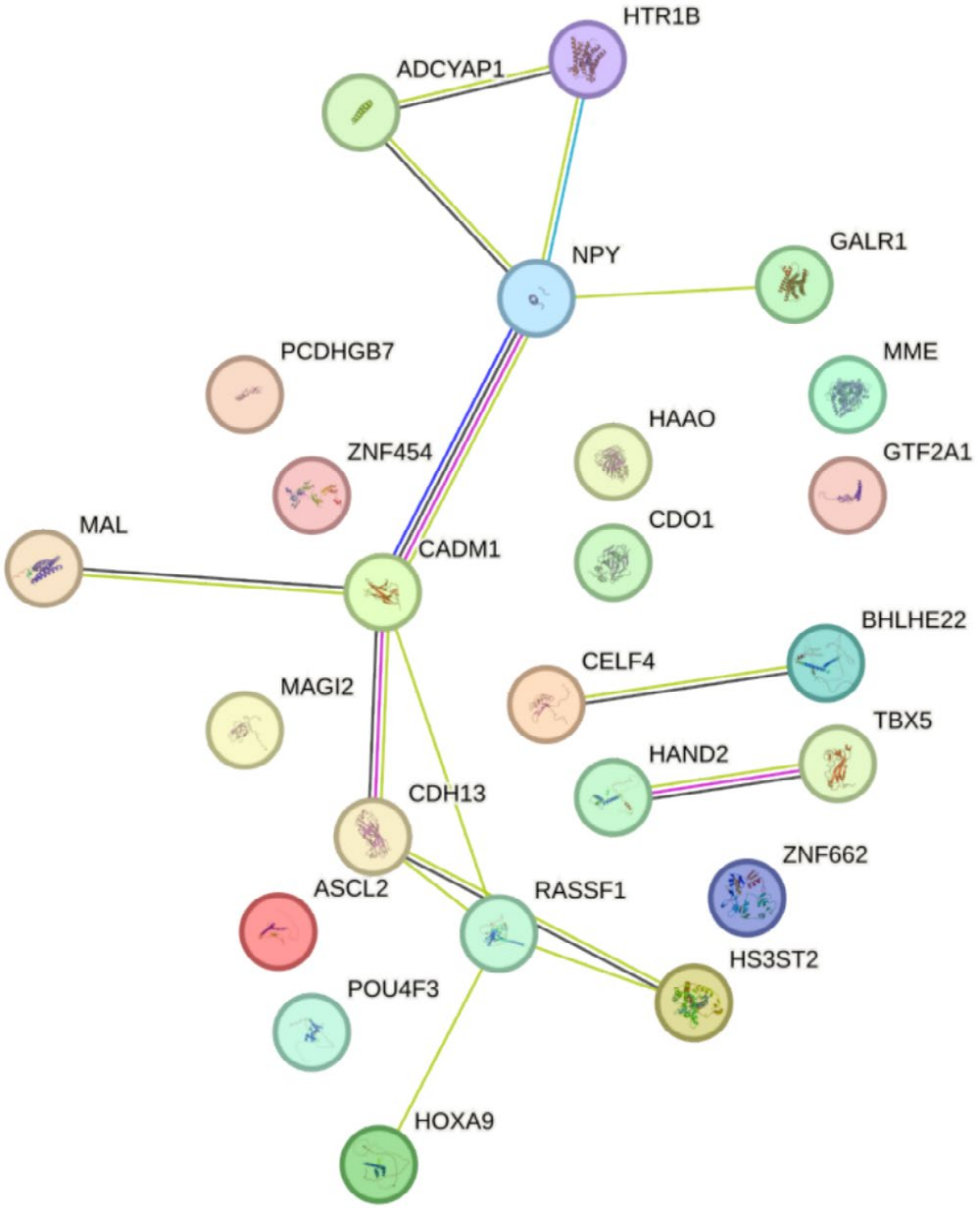
Interactions of the methylation‐related gene shown by the PPI network (score = 0.453).

To examine the interactions among genes associated with methylation, we conducted a PPI analysis, utilizing a minimum interaction score threshold of 0.453, which corresponds to a medium confidence level. The analysis identified CDH13, HS3ST2, CADM1, HTR1B, NPY, and RASSF1 as core genes, each exhibiting a node degree greater than 10. (Figure 1). The *p* value for PPI enrichment is 1.16 × 10^−8^.

### 3.2 The Correlation Between the Occurrence and Development of Endometrial Cancer

#### 3.2.1 Methylation Expression in Normal Tissue and Tumor Tissue

**FIGURE 2:**
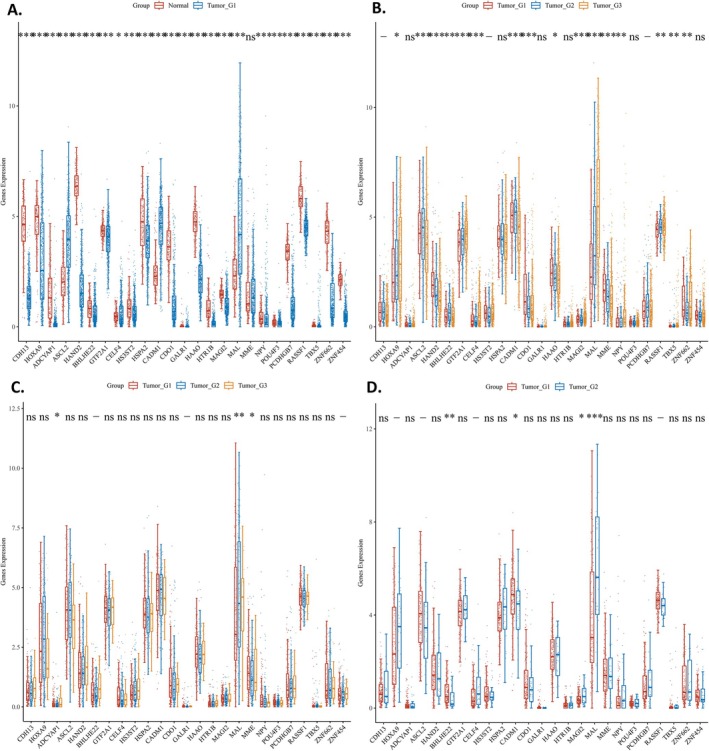
(A) The expression distribution of Methylation related gene in tumor tissues and normal tissues. (B) The expression distribution of Methylation‐related gene in G1, G2, G3. (C) The expression distribution of Methylation‐related gene in IA, IB, IC. (D) The expression distribution of Methylation‐related gene in IA and IIIC.

The expression distribution of 25 genes was analyzed in both tumor and normal tissues. Among these genes, the methylation status of the MME gene did not exhibit a significant difference between tumor and normal tissues. In contrast, the methylation levels of the remaining genes demonstrated a statistically significant difference between the two tissue types (*p* < 0.05) (Figure 2A).

#### 3.2.2 Expression of Methylation‐Related Genes in Tumors With Different Differentiation

The expression distribution of 25 genes in groups G1, G2, and G3 was analyzed. Among these 25 genes, the methylation levels of HOXA9, ASCL2, HAND2, BHLHE22, GTF2A1, CELF4, CADM1, CDO1, HAAO, MAGI2, MAL, MME, NPY, RASSF1, TBX5, and ZNF662 exhibited significant differences (*p* < 0.05) (Figure 2B).

#### 3.2.3 Expression of Methylation‐Related Genes in Patients With Different Invasion

The expression distribution of 25 genes in groups IA, IB, and IC was analyzed. Among these 25 genes, the methylation levels of ADCYAP1, MAL, and MME exhibited statistically significant differences (*p* < 0.05) (Figure 2C).

#### 3.2.4 Expression of Methylation‐Related Genes Between Patients With Retroperitoneal Lymph Node Metastasis or Not

The expression distribution of 25 genes in IA and IIIC was analyzed. Among these genes, the methylation levels of BHLHE22, CADM1, MAGI2, and MAL exhibited statistically significant differences (*p* < 0.05) (Figure 2D).

#### 3.2.5 Construction of the MRGs Prognostic Signature in TCGA Cohort

**FIGURE 3:**
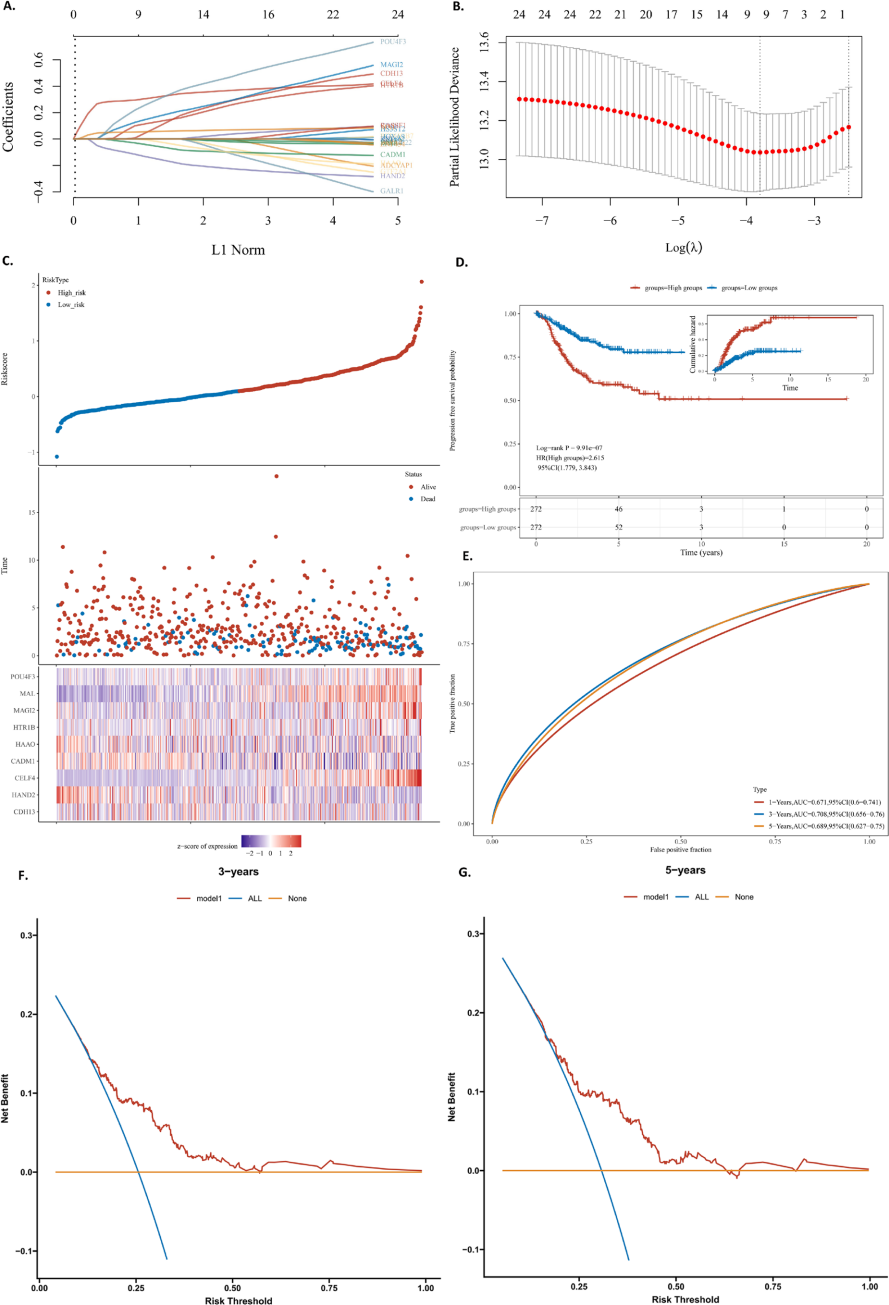
(A) LASSO model was adjusted based on the minimum criteria (regularization parameter λ). (B) The optimal log value of lambda was indicated by the first black dotted line from the left. (C) Kaplan–Meier curve analyses for the high‐risk group and low‐risk group were classified according to the median risk score (RS). And heat map presented expression profiles of risk genes in prognostic models for high‐ and low‐risk groups. (D) Kaplan–Meier curve of progression‐free survival (PFS) for EC samples. (E) AUC curve evaluating the predictive performance of the risk signature. (F, G) Decision curve analysis of candidate mRNAs for predicting survival status.

A recurrence prediction model using LASSO Cox regression was developed from methylation‐related genes in the TCGA training cohort, with independent variable coefficients shown in Figure 3A. Based on the optimal log value of lambda, we identified nine genes (Figure 3C): CDH13, HAND2, CELF4, CADM1, HAAO, HTR1B, MAGI2, MAL, and POU4F3. A risk score (RS) was calculated using multivariate Cox regression analysis to represent a comprehensive index of the methylation‐related gene signature. This was achieved by multiplying the expression level of each gene by its corresponding coefficient (with lambda.min = 0.0223), as follows:

Riskscore = (0.0757) *CDH13 + (−0.1451) *HAND2 + (0.3138) *CELF4 + (−0.0508) *CADM1 + (−0.0145) *HAAO+(0.1255) *HTR1B+(0.1627) *MAGI2 + (0.0565) *MAL+(0.2852) *POU4F3.

The expression of nine genes was identified with significant differences between high‐risk and low‐risk groups. EC samples in the TCGA training cohort were divided into high‐ and low‐risk groups with the median RS as the threshold. The RS distribution and its correlation with recurrence outcomes between the two groups were shown in a scatter plot, and the expression profiles of risk genes in prognostic models for both groups were shown in a heat map (Figure 3C). The Kaplan–Meier curve indicates that EC samples with elevated RS are associated with a higher risk of recurrence (Figure 3D). The Area Under the Curve (AUC) values of the recurrence prediction model were 0.671, 0.708, and 0.689 for the 1‐year, 3‐year, and 5‐year time points, respectively (Figure 3E), indicating a robust prediction capability. The Decision Curve Analysis (DCA) curve surpasses the “all” and “none” curves (Figure 3F,G), showing that the model based on methylation‐related genes excels in robustness and predictive accuracy.

**FIGURE 4:**
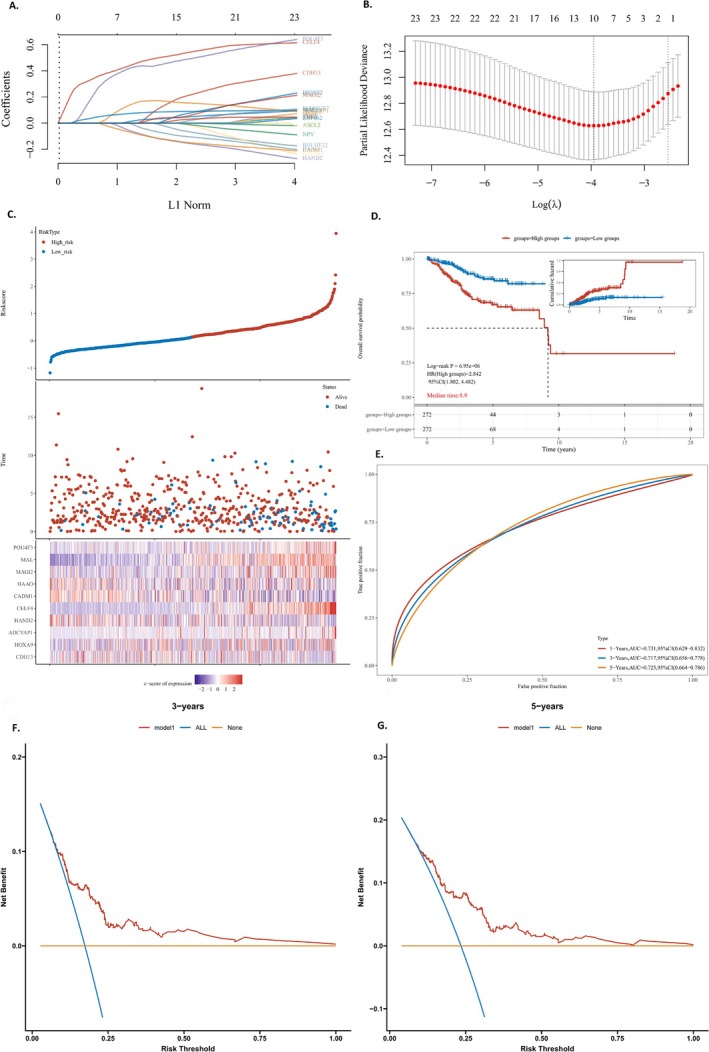
(A) LASSO model was adjusted based on the minimum criteria (regularization parameter λ). (B) The optimal log value of lambda was indicated by the first black dotted line from the left. (C) Kaplan–Meier curve analyses for the high‐risk group and low‐risk group were classified according to the median risk score (RS). And heat map presented expression profiles of risk genes in prognostic models for high‐ and low‐risk groups. (D) Kaplan–Meier curve of overall survival (OS) for EC samples. (E) AUC curve evaluating the predictive performance of the risk signature. (F, G) Decision curve analysis of candidate mRNAs for predicting survival status.

A prognostic model using LASSO Cox regression was created based on prognosis‐related methylation genes with the TCGA training cohort, with coefficients shown in Figure 4A. Utilizing the optimal log value of lambda, we identified 10 genes (Figure 4B): POU4F3, MAL, MAGI2, HAAO, CADM1, CELF4, HAND2, ADCYAP1, HOXA9, and CDH13. With multivariate Cox regression analysis, an RS representing the comprehensive index of the methylation‐related gene signature was calculated. This was achieved by multiplying the expression level of each gene by its corresponding coefficient(lambda.min = 0.0192).

Risk score = (0.0142) *CDH13 + (0.0257) *HOXA9 + (0.1669) *ADCYAP1 + (−0.0862) *HAND2 + (0.4718) *CELF4 + (−0.0922) *CADM1 + (−0.0382) *HAAO+(0.0056) *MAGI2 + (0.0799) *MAL+(0.4377) *POU4F3.

The expression of ten genes was also identified with significant differences between high‐risk and low‐risk groups. The RS and prognosis were further investigated between high‐risk and low‐risk groups with the median RS as the threshold. The RS distribution and its correlation with survival outcomes were shown in a scatter plot (Figure 4C). The expression profiles of genes within prognostic‐related models were shown with a heat map for both high‐ and low‐risk groups (Figure 4C). Furthermore, the Kaplan–Meier survival analysis indicates that EC samples with elevated RS are associated with a poorer prognosis (Figure 4D). The AUC values of the prognostic‐related model were 0.731, 0.717, and 0.725 for the 1‐, 3‐, and 5‐year intervals, respectively (Figure 4E), indicating a strong predictive capability. The decision curve analysis (DCA) curve was positioned above the “all” and “none” curves (Figure 4F,G). These findings suggest that the model, based on methylation‐related genes, has a high performance in terms of robustness and predictive accuracy (Table 1).

**TABLE 1:** Summary of key predictive performance metrics.

Metric	1‐year AUC	3‐year AUC	5‐year AUC
Recurrence model	0.671	0.708	0.689
Prognostic model	0.731	0.717	0.725

#### 3.2.6 Identification of EC Molecular Subtypes Based on NMF Algorithm

**FIGURE 5:**
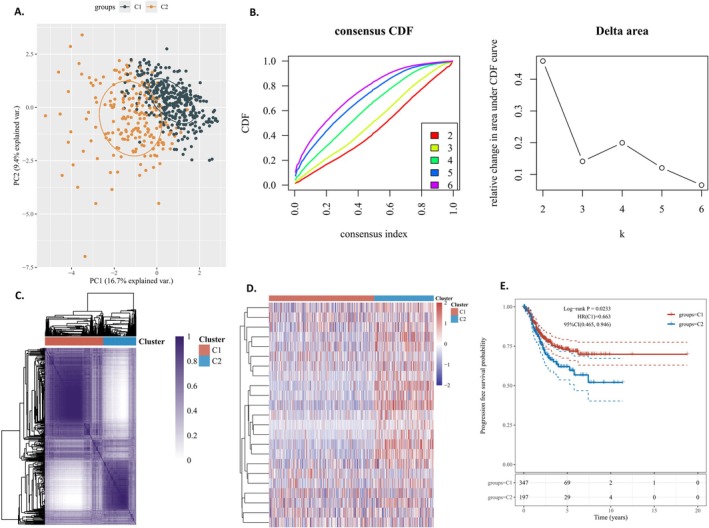
(A) The PCA scatter plot indicated a reduced overlap in the sample distributions of Group 1 and Group 2. (B) Consensus clustering cumulative distribution function (CDF) and relative change in the area under the CDF curve (CDF Delta area). (C) Heatmap of nsNMF consensus matrix of *K* = 2. (D) Unsupervised clustering of immune‐ and methylation‐related gene expression profiling between the two clusters. *p < 0.05, **p < 0.01, ***p < 0.001. ns, no significance. (E) Kaplan–Meier curve of progression‐free survival (PFS) for EC subtypes.

The PCA scatter plot showed less overlap between Group 1 and Group 2 sample distributions, with clearly distinct clustering distances that matched the expected effect (Figure 5A). Figure 5B shows the cumulative distribution function (CDF) for different cluster counts (k). A higher CDF value indicates more robust clustering for that k. The Delta area plot in Figure 5B displays the change in the area under the CDF curve from k‐1 to k, with a larger Delta suggesting a notable improvement in clustering quality. Combining these plots helps identify the best k value by ensuring a smoother, higher CDF distribution with a larger area under the curve, as shown by the inflection point method. At *k* = 2, the CDF is smoother and nearly maximal, suggesting optimal clustering results.

Two molecular subtypes were identified using the NFM clustering algorithm based on methylation‐related genes (Figure 5C). Figure 5D shows unsupervised clustering of differential expression profiles between these subtypes. The Kaplan–Meier curve indicates that group 2 had significantly worse progression‐free survival (PFS) than group 1 (Figure 5E).

### 3.3 Treatment Response of Different Drugs

#### 3.3.1 Correlated of Methylation Gene Expression and Hormone Therapy Responses in EC

**FIGURE 6:**
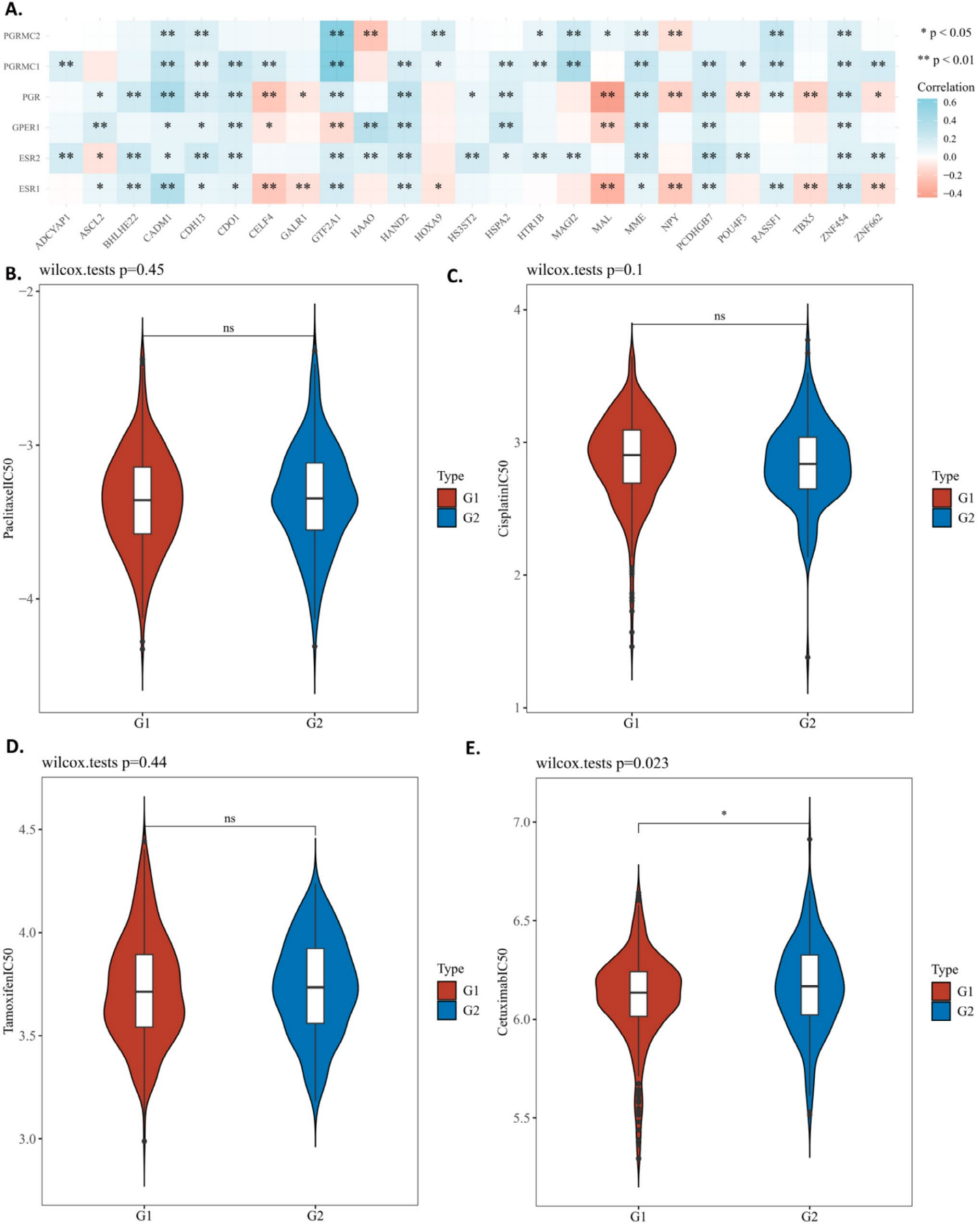
(A) A heatmap of the correlation between methylation‐related genes and Estrogen receptor‐related genes. (B) The distribution of IC50 scores of paclitaxel. (c) The distribution of IC50 scores of cisplatin. (D) The distribution of IC50 scores of tamoxifen. (E) The distribution of IC50 scores of cetuximab.

The Spearman correlation analysis revealed a significant link between ER/PR‐related genes and methylation‐related genes (Figure 6A). Using the Genomics of Drug Sensitivity in Cancer (GDSC) database, the response of EC to tamoxifen in TCGA datasets was assessed. TCGA EC patients were divided into two cohorts: 347 low‐risk and 197 high‐risk recurrence patients. No difference was observed(Figure 6D).

#### 3.3.2 Correlated of Methylation‐Related Gene Expression and Chemotherapeutic Responses in EC

The response of endometrial cancer (EC) to paclitaxel, cisplatin, and cetuximab was evaluated between Group 1 and Group 2 using data from the Genomics of Drug Sensitivity in Cancer (GDSC) database. The findings reveal that patients in Group 1 exhibited a lower half‐maximal inhibitory concentration (IC50) and demonstrated increased sensitivity to cetuximab compared to patients in Group 2 (Figure 6E). In contrast, no significant differences in IC50 values for cisplatin and paclitaxel were detected between the two groups (Figure 6B,C). These results suggest a potential therapeutic benefit of cetuximab for patients in Group 1.

#### 3.3.3 Predictive Role of the MRGs in Predicting Response to Immunotherapy

To further explore the influence of the methylation‐related gene prognostic signature on immunotherapy efficacy, we examined the associations between the risk score (RS) and immune infiltration within the tumor microenvironment (TME). The assessment of the TME was performed utilizing three distinct analytical approaches: TIMER, MCPcounter, and CIBERSORT.

**FIGURE 7:**
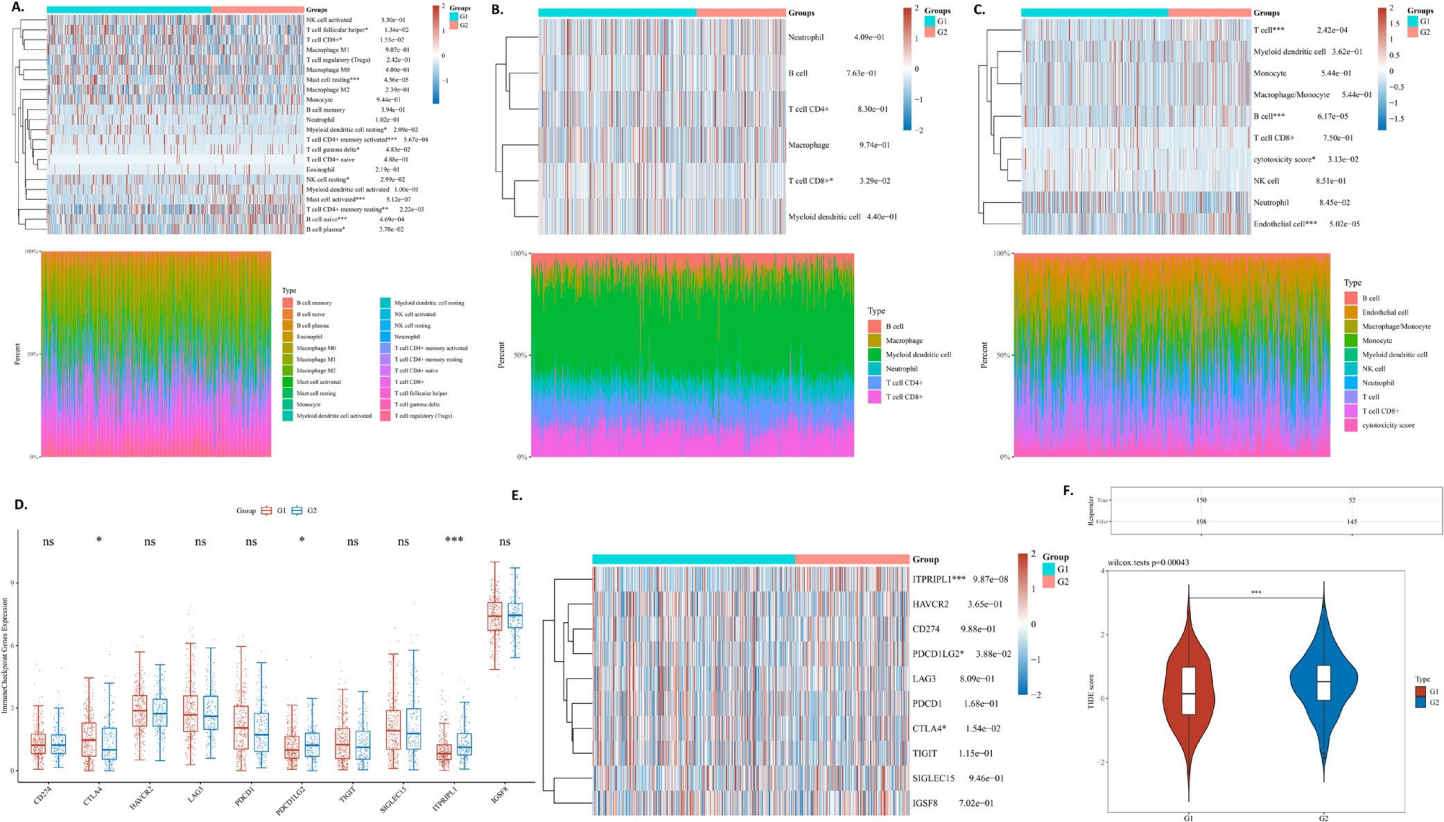
(A) The expression distribution of CIBERSORT immune score in G1 and G2. (B) The expression distribution of TIMER immune score in G1 and G2. (C) The expression distribution of MCPCOUNTER immune score in G1 and G2. (D, E) Correlation of the RS and immune checkpoint expressions. (F) Statistical table of immune response of samples in different groups in the prediction results, and the distribution of immune response scores in different groups in the prediction results.

In the TIMER mode, as illustrated in Figure 6A, CD8+ T cells in Group 2 were significantly decreased (Figure 7B). Conversely, in the MCPCOUNTER mode, as depicted in Figure 7C,T cells, B cells, and endothelial cells in Group 2 were significantly increased. Additionally, the cytotoxicity scores were significantly decreased. In the CIBERSORT analysis, we observed significantly higher proportions of naive B cells, plasma B cells, and resting memory CD4 T cells, as well as activated mast cells and gamma delta T cells in Group 2. Conversely, Group 2 exhibited significantly lower proportions of activated memory CD4 T cells, CD8 T cells, follicular helper T cells, resting NK cells, resting mast cells, and myeloid dendritic cells resting (Figure 6A).

We also investigate the expression of immune checkpoints‐related genes (CD274, CTLA4, HAVCR2, LAG3, PDCD1, PDCD1LG2, TIGIT, SIGLEC15, ITPRIPL1, IGSF8) between two groups. Significant differences were observed in the expression levels of immune checkpoint genes such as CTLA4, PDCD1LG2, and ITPRIPL1 between Group 1 and Group 2 (Figure 7D, Figure 7E). In Group 1, the expression of CTLA4 was significantly elevated, whereas the expression levels of PDCD1LG2 and ITPRIPL1 were significantly reduced. The data suggest that the methylation‐related gene signature may contribute to evaluating the response to immunotherapy in patients with EC. Further analysis showed that Group 2 exhibits a higher TIDE score than Group 1 and benefits less from ICB treatment (Figure 7F) [16].

#### 3.3.4 Methylation‐Related Gene Evolution After Endocrine Treatment Exposed

**FIGURE 8:**
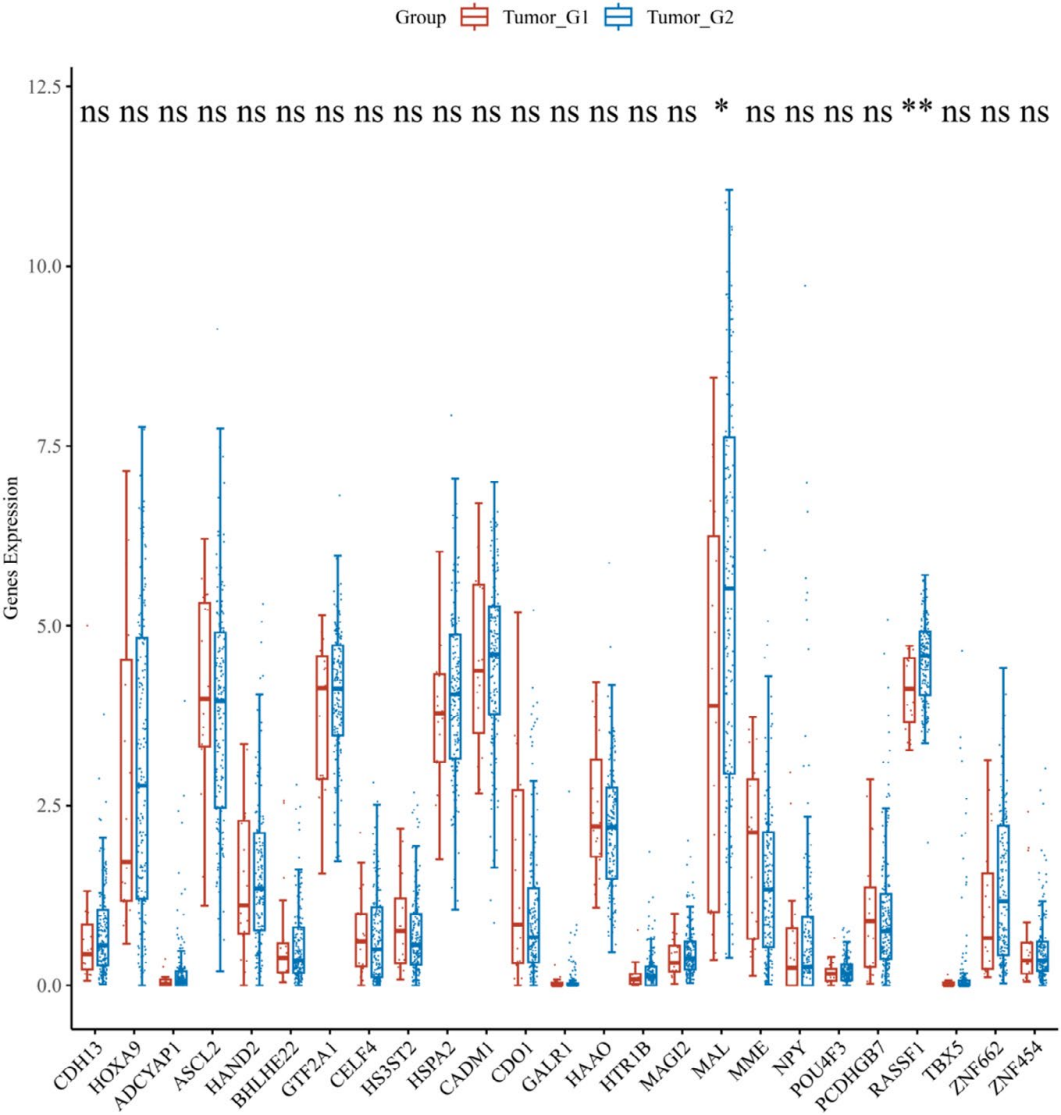
The expression distribution of Methylation‐related gene between the endocrine therapy group and the chemotherapy group.

Endocrine therapy constitutes a pivotal intervention for advanced or metastatic endometrial cancer (EC). To investigate alterations in methylation‐related gene expression subsequent to endocrine therapy, we performed a comparative analysis of the expression levels of methylated genes between the endocrine therapy cohort (encompassing both monotherapy and combination therapy with chemotherapy) and the chemotherapy cohort. The findings revealed a significant downregulation of MAL and RASSF1 following endocrine treatment (Figure 8).

## 4 Discussion

Numerous models were developed to predict the recurrence and treatment response of different cancer types [16, 17, 18, 19, 20]. However, no promising prediction model of EC was universally accepted in the management of EC. In this study, we identified 25 genes associated with methylation. Significant differences were observed in overall survival (OS), progression‐free survival (PFS), molecular characteristics, tumor microenvironment, sensitivity to immunotherapy, and sensitivity to chemotherapy among the various methylation‐related genes. Based on methylation‐related genes, we developed a risk model to predict patient prognosis and treatment response to different drugs. Through LASSO Cox regression analysis, we identified CDH13, HAND2, CELF4, CADM1, HAAO, HTR1B, MAGI2, MAL, POU4F3, ADCYAP1, and HOXA9 as significant predictors of endometrial cancer prognosis. Among these, we identified 11 genes (CDH13, HAND2, CELF4, CADM1, HAAO, HTR1B, MAGI2, MAL, POU4F3, ADCYAP1, HOXA9) and constructed a risk model using LASSO regression analysis. We calculated the risk scores and stratified patients into high‐risk and low‐risk groups accordingly. The high‐risk group exhibited a poorer prognosis compared to the low‐risk group. The ROC curve and internal dataset validation demonstrated the robust predictive performance of this model. Furthermore, the model outperformed certain clinical indices, such as disease stage, in predicting patient prognosis. Compared to traditional Cox regression models, LASSO regression offers advantages in variable selection and overfitting prevention. Our model demonstrated superior predictive performance over clinical staging (AUC 0.73 vs. 0.65), aligning with recent studies on methylation‐based prognostic tools.

The genes POU4F3, MAL, MAGI2, HAAO, CADM1, CELF4, HAND2, ADCYAP1, HOXA9, CDH13, and HTR1B play pivotal or potential pivotal roles in EC through epigenetic dysregulation, influencing key cellular processes such as proliferation, differentiation, and metastasis. POU4F3, a transcription factor involved in neuronal differentiation, is often hypomethylated in EC, leading to its overexpression and promoting tumor cell survival and resistance to apoptosis [21]. Similarly, HAAO and ADCYAP1, which regulate metabolic and angiogenic pathways, are frequently hypomethylated, enhancing tumor growth and immune evasion [22]. On the other hand, hypermethylation of tumor suppressor genes such as MAL, MAGI2, CADM1, HOXA9, and CDH13 silences their expression, disrupting cell adhesion, polarity, and differentiation. For instance, MAL hypermethylation compromises cell polarity, while CADM1 silencing facilitates metastasis and chemoresistance. These epigenetic alterations collectively destabilize cellular homeostasis and drive EC progression [14].

The functional roles of these genes extend to critical signaling pathways. MAGI2 hypermethylation disrupts Wnt/β‐catenin signaling, promoting tumor invasiveness [23], while HAND2 hypermethylation impairs progesterone signaling, contributing to hormone therapy resistance [24]. CELF4, an RNA‐binding protein, is overexpressed due to hypomethylation, leading to aberrant splicing events that drive tumor proliferation [25]. Additionally, HTR1B hypomethylation activates serotonin‐mediated MAPK/ERK signaling, further enhancing cell proliferation and invasion [22]. These findings highlight the complex interplay between epigenetic modifications and oncogenic signaling in EC, underscoring the importance of methylation patterns in tumor biology.

The clinical implications of these epigenetic alterations are profound. Methylation profiles of these genes could serve as valuable biomarkers for early diagnosis, prognosis, and therapeutic stratification. For example, CDH13 hypermethylation is associated with advanced disease and poor survival [26], while HTR1B hypomethylation may predict responsiveness to serotonin pathway inhibitors [22]. Targeting these methylation‐driven alterations, either through epigenetic modifiers (e.g., DNMT inhibitors) or downstream effectors (e.g., VEGF inhibitors for ADCYAP1), offers promising therapeutic avenues. Future research should focus on validating these biomarkers in larger cohorts and exploring combinatorial therapies that address both epigenetic and molecular vulnerabilities in EC, ultimately advancing precision oncology approaches for this malignancy.

TCGA molecular subtypes have been used to predict the immune response of EC. Studies show that 20%–30% of patients with advanced EC could benefit from the approved ICB, such as dostarlimab and pembrolizumab [27, 28]. In this study, the immune cells in the TME were analyzed with three different computational tools: TIMER, MCP‐counter, and CIBERSORT. In the TIMER mode, CD8+ T cells were significantly decreased in patients with low‐risk scores. The MCP‐counter mode assesses more cell types and found the cytotoxicity scores increased significantly in this population. In the CIBERSORT analysis with 22 cell types, patients in the low risk group showed a higher proportion of naive B cells, plasma B cells, resting memory CD4 T cells, activated mast cells, and gamma delta T cells, but a significantly lower proportion of activated memory CD4 T cells, CD8 T cells, follicular helper T cells, resting NK cells, resting mast cells, and myeloid dendritic cells This study found that patients in the high risk group showed a higher TIDE scores than those in the low‐risk group and may benefit more from ICB treatment, while patients in the low risk group with high CTLA4 expression may benefit from anti‐CTLA4 treatment. Besides, the expression of PDCDILG2 (PD‐L2) and ITPRIPL1 was upregulated in patients of the high‐risk group, which may benefit from PD‐1 inhibitors [29, 30]. ITPRIPL1 is an uncharacterized type I membrane protein that inhibits the function of CTLs, especially in tumors with low PD‐L1 expression. The extracellular domain of ITPRIPL1 directly binds to the CD3ε extracellular domain, inhibiting TCR‐CD3 signaling and hindering the initial activation of T cells, thus promoting tumor immune escape and progression [31, 32]. Targeting ITPRIPL1 may be an alternative treatment for patients in the high risk group. A combination of targeted treatment of methylation‐related genes and immune checkpoint inhibitors may also be a novel selection for EC therapy. Cetuximab is an IgG1 monoclonal antibody that targets the epidermal growth factor receptor. Findings from a Phase II clinical trial indicated that cetuximab treatment in patients with progressive or recurrent esophageal cancer demonstrated good tolerability and yielded a clinical benefit rate of 15% [33]. Our study found that cetuximab was more sensitive to patients in the low risk group based on MRGs.

While our model demonstrates strong predictive performance, its retrospective design and reliance on TCGA data necessitate validation in prospective cohorts. Additionally, the biological roles of understudied genes like ITPRIPL1—a novel immune evasion marker [31]—warrant experimental validation. Future studies should explore dynamic methylation changes during treatment, particularly in response to epigenetic therapies (e.g., DNMT inhibitors), and assess the model's utility in guiding combination therapies (e.g., ICIs + cetuximab).

In conclusion, the model based on the methylation‐related genes showed promising outcomes in predicting the recurrence and treatment response of EC, which provides a novel biomarker in the management of EC. The patients with high‐risk scores showed a poorer prognosis and may benefit more from the treatment of cetuximab or immune checkpoint inhibitors. Further studies are needed to verify the model in the clinics.

## Author Contributions

R.R. and M.W. conceived and directed the completion of the study. M.W. collected and downloaded the data. R.R. conducted the data analysis and drafted the manuscript. M.W. and J.M. edited the manuscript. All authors contributed to the manuscript and approved the submitted version.

## Ethics Statement

This article does not contain any studies with human participants or animals performed by any of the authors. All data involved in this study come from public databases.

## Conflicts of Interest

The authors declare no conflicts of interest.

## Supporting information


**Table S1.** Methylation‐related genes of previous study.

## Data Availability

Research data are not shared.
