# Supplementary material for: The Prognosis Prediction Model for Endometrial Cancer Based on DNA Methylation Signature
Source: Cancer Rep (Hoboken). 2025 Jun 17;8(6):e70218. doi: 10.1002/cnr2.70218 (PMC12174473; doi:10.1002/cnr2.70218)
Supplement: Supplementary file 1 — Table S1. Methylation‐related genes of previous study. [file CNR2-8-e70218-s001.docx]

**Supplementary Table 1** methylation-related genes of previous study

| **Methylation related gene** | **Gene function** | **Study (year)** | **Ref.** |
| --- | --- | --- | --- |
| CDH13 | Regulation at the DNA and chromosome levels | Bakkum-Gamez (2015)，Wentzensen (2014)，Birgit M.（2023） | 1,2,3 |
| HOXA9 | Regulation at the DNA and chromosome levels | Bakkum-Gamez (2015)  Sangtani (2020) | 1,4 |
| ADCYAP1 | Regulation of transcription levels | Bakkum-Gamez (2015)，Wentzensen (2014) | 1,2 |
| ASCL2 | Regulation of transcription levels | Bakkum-Gamez (2015) | 1 |
| HAND2 | Regulation of transcription levels | Jones (2013)  Liew (2019)  Birgit M.（2023） | 3,5,6 |
| BHLHE22 | Regulation of transcription levels | Birgit M.（2023) | 3 |
| GTF2A1 | Regulation of transcription levels | Bakkum-Gamez (2015) | 1 |
| CELF4 | Post-transcriptional regulation | Huang (2017)，Kong2023 | 7,8 |
| HS3ST2 | The regulation of translation level | Wentzensen (2014)  Bakkum-Gamez (2015) | 1,2 |
| HSPA2 | The regulation of translation level | Bakkum-Gamez (2015) | 1 |
| CADM1 | Post-translational regulation | De Strooper (2014) | 2 |
| CDO1 | Post-translational regulation | Wang（2022), Liew (2019),Huang (2017), Birgit M.（2023），Kong2023， | 3,6,7,8,9 |
| GALR1 | Post-translational regulation | Birgit M.（2023），Doufekas (2013)， | 3,10 |
| HAAO | Post-translational regulation | Bakkum-Gamez (2015) | 1 |
| HTR1B | Post-translational regulation | Bakkum-Gamez (2015)，Wentzensen (2014), Sangtani (2020)， | 1,2,4 |
| MAGI2 | Post-translational regulation | Chang (2018) | 11 |
| MAL | Post-translational regulation | De Strooper (2014) | 12 |
| MME | Post-translational regulation | Bakkum-Gamez (2015)，Wentzensen (2014) | 1,2 |
| NPY | Post-translational regulation | Bakkum-Gamez (2015)，Wentzensen (2014) | 1,2 |
| POU4F3 | Regulation of transcription levels | Chang (2018) | 11 |
| PCDHGB7 | Post-translational regulation | Yuan（2022） | 13 |
| RASSF1 | Post-translational regulation | Bakkum-Gamez (2015)，Sangtani (2020) | 1,4 |
| TBX5 | Regulation of transcription levels | Liew (2019) | 6 |
| ZNF662 | Regulation of transcription levels | Huang (2017) | 7 |
| ZNF454 | Regulation of transcription levels | Wang（2022） | 9 |

**References for methylation-related genes**

1. Bakkum-Gamez JN, Wentzensen N, Maurer MJ, Hawthorne KM, Voss JS, Kroneman TN, et al. Detection of endometrial cancer via molecular analysis of DNA collected with vaginal tampons. Gynecol Oncol. 2015;137:14-22.

2. Wentzensen N, Bakkum-Gamez JN, Killian JK, Sampson J, Guido R, Glass A, et al. Discovery and validation of methylation markers for endometrial cancer. Int J Cancer. 2014;15;135:1860-8.

3. Wever BMM, van den Helder R, van Splunter AP, van Gent MDJM, Kasius JC, Trum JW, et al. DNA methylation testing for endometrial cancer detection in urine, cervicovaginal self-samples, and cervical scrapes. Int J Cancer. 2023;153:341-351.

4. Sangtani A, Wang C, Weaver A, Hoppman NL, Kerr SE, Abyzov A, et al. Combining copy number, methylation markers, and mutations as a panel for endometrial cancer detection via intravaginal tampon collection. Gynecol Oncol. 2020;156:387-392.

5. Jones A, Teschendorff AE, Li Q, Hayward JD, Kannan A, Mould T, et al. Role of DNA methylation and epigenetic silencing of HAND2 in endometrial cancer development. PLoS Med. 2013;10:e1001551.

6. Liew PL, Huang RL, Wu TI, Liao CC, Chen CW, Su PH, et al. Combined genetic mutations and DNA-methylated genes as biomarkers for endometrial cancer detection from cervical scrapings. Clin Epigenetics. 2019;11:170.

7. Huang RL, Su PH, Liao YP, Wu TI, Hsu YT, Lin WY, et al. Integrated Epigenomics Analysis Reveals a DNA Methylation Panel for Endometrial Cancer Detection Using Cervical Scrapings. Clin Cancer Res. 2017;23:263-272.

8. Kong LH, Xiao XP, Wan R, Chao XP, Chen XJ, Wang J. The role of DNA methylation in the screening of endometrial cancer in postmenopausal women. Zhonghua Yi Xue Za Zhi. 2023;103:907-912.

9. Wang L, Dong L, Xu J, Guo L, Wang Y, Wan K, ,et al. Hypermethylated CDO1 and ZNF454 in Cytological Specimens as Screening Biomarkers for Endometrial Cancer. Front Oncol. 2022;12:714663.

10. Doufekas K, Hadwin R, Kandimalla R, Jones A, Mould T, Crowe S, et al. GALR1 methylation in vaginal swabs is highly accurate in identifying women with endometrial cancer. Int J Gynecol Cancer. 2013;23:1050-5.

11. Chang CC, Wang HC, Liao YP, Chen YC, Weng YC, Yu MH, et al. The feasibility of detecting endometrial and ovarian cancer using DNA methylation biomarkers in cervical scrapings. J Gynecol Oncol. 2018;29:e17.

12. De Strooper LM, van Zummeren M, Steenbergen RD, Bleeker MC, Hesselink AT, Wisman GB, et al. CADM1, MAL, and miR124-2 methylation analysis in cervical scrapes to detect cervical and endometrial cancer. J Clin Pathol. 2014;67:1067-71.

13. Yuan J, Mao Z, Lu Q, Xu P, Wang C, Xu X, et al. Hypermethylated PCDHGB7 as a Biomarker for Early Detection of Endometrial Cancer in Endometrial Brush Samples and Cervical Scrapings. Front Mol Biosci. 2022;8:774215.
